# Drivers of business model innovation in micro and small enterprises: evidence from Egypt as an emerging economy

**DOI:** 10.1186/s43093-022-00180-2

**Published:** 2023-02-18

**Authors:** Rasha A. A. ElNaggar, Mayar Farrag ElSayed

**Affiliations:** 1 Faculty of Commerce, Helwan University, Cairo, Egypt; 2 Faculty of economics and international trade, Egyptian Chinese Universiry, Cairo, Egypt

**Keywords:** Business model innovation, Social capital, Market orientation, Environmental uncertainty, Networking, Resilience strategies, Micro enterprises, Small enterprises, Political ties, Business ties

## Abstract

Despite its recognition as a resilience strategy, there is a scarcity of research on business model innovation (BMI) in micro and small enterprises in emerging economies. Business model innovation drivers in micro and small enterprises may differ from those in large firms in developed economies. In micro and small businesses, BMI is determined by the dynamic capabilities of the business, as well as the ability of the business owner to reconfigure and exploit its resources in a way that creates market value. This is one of few studies that connects the concepts of social capital and  market orientation to explain business model innovation and its consequences in a turbulent business environment. This study investigates the impact of managerial ties, business owner market orientation and perceived environmental uncertainty as drivers to BMI. Also, it investigates the effect of BMI on business performance. The results of analyzing the responses of 426 micro and small enterprises by Smart PLS 3.3 show that business model innovation mediates the relationship between business ties and business performance. Surprisingly, the results proved that micro and small business owners adopting reactive market orientation, contribute to BMI more than those adopting proactive market orientation. The findings emphasize the importance of micro and small business owner in driving BMI. This study ensures that BMI is a function of how resources can be deployed and configured dynamically to generate value.

## Introdution

Since the outbreak of COVID-19, the global business climate has been disrupted; its dynamism has changed, making it difficult to forecast. It was the beginning that caused global supply chain disruption. Political tensions between Ukraine, Russia, and the USA have also had an impact on the global business environment. In addition to global uncertainties, enterprises may face threats and risks as a result of domestic government policy. The risk buffers available to large corporations may not be accessible to micro and small enterprises [31, 32, 48, 55].

The majority of countries recognize the importance of SMEs in economic development [55]. Micro, small, and medium-sized enterprises (SMEs) are regarded as strategic partners in the development of emerging economies, including Egypt's. According to the Central Agency for Public Mobilization and Statistics (CAPMAS), there are 67,000 small businesses and 3–8 million micro enterprises in 2020, totaling $4.9 billion [19]. Since the 1990s, there has been a significant increase in the number of SMEs in Egypt; it has been providing job opportunities to compensate for the decline in public-sector job opportunities, accounting for 75% of private-sector employment. Micro and small businesses represent 91% of all businesses in the Egyptian economy; the SMEs represent 80% of Egypt's GDP; 75% of private sector employment and 99% of non-agriculture employment [87].

In the late 1990s and early 2000s, the Egyptian government enacted the "National Policy for SME Development" to assist SMEs. Since then, the Egyptian government adopted entrepreneurship as a strategy and a practice [1]. Under the Entrepreneur National Strategy adopted by the Egyptian governmental institutions, a variety of programs were offered to support and finance the expansion of SMEs. As a result, other pieces of legislations were launched to offer SMEs the financial and the technical assistance securing their sustainable development. For example, the Central Bank of Egypt has established the SMEs-funding initiative (2016–2019) to finance SMEs. To fully capitalize on available assistance and turn it into a competitive advantage, SMEs should adopt innovative practices that allow them to successfully adapt to a changing business environment. Because of its ability to generate internal and external competitive advantage, innovation is becoming a critical business strategy that improves business performance [48, 53].

Innovative activities are not only crucial for SMEs' competitiveness, but they also contribute to their sustainable success [7]. The ability of micro and small businesses to reconfigure their existing limited resources, overcome risks, seize opportunities, and challenge the status quo by implementing workable business model innovation determines their resilience in a changing uncertain business environment [18].

While most studies discussed business model innovation (BMI) in large organizations, only few have focused on micro and small enterprises [66]. Micro and small businesses differ from large corporations in that the role of the business owner is more visible and influential in driving the overall micro and small business strategy. This may limit the transferability of large firm practices to micro and small businesses [63]. Unlike product and technology innovation, that can be replicated by competitors once released, business model innovation may be difficult for competitors to replicate because it must strongly align with the business’s key competencies.

The firm's dynamic capabilities determine the speed, degree, and associated costs of business model innovation (BMI) required to produce and deliver value to stakeholders. Similarly, a company's dynamic capabilities determine its ability to innovate and determine the most appropriate business model architecture [55]. Accordingly, copying BMI from large to micro and small enterprises may be unreasonable. Small and micro enterprise literature requires continuous updating for those dynamic capabilities that contribute to business model innovation [42, 86]. Given the importance of micro and small businesses in Egypt's socioeconomic development as an emerging economy, understanding and identifying micro and small business resilience strategies is becoming increasingly important. This study is one of the few in the field of micro and small businesses that addresses business model innovation, a well-established resilience strategy, and identifies its antecedents and consequences. In several ways, this study contributes to the understanding of BMI in micro and small enterprises. First, this study identifies the mix of dynamic capabilities that enables micro and small businesses in Egypt to innovate their business models. Second, it is one of the few studies that looks into the impact of micro and small business owners' market orientation on BMI. Third, this study provides a road map for implementing BMI, one of the resilience strategies.

### Theoretical background and hypotheses development

In unstable business environments, regardless of firm size, innovation is becoming increasingly important for firm survival. These innovations might range from simple incremental to complicated radical ones. Unlike large corporations, micro and small enterprises may lack the resources, time, and funds needed to pursue innovations that sustain competitive advantage [40].

The relevance of company resources and capabilities as primary drivers of innovation has been discussed by two related theories: the resource-based and the dynamic capabilities theories. Although the resource-based theory recognizes the importance of valuable and scarce resources in achieving competitiveness, it provides few details on how organizations can broaden their pool of internal and external competencies and develop new capabilities in changing business environments [52]. Dynamic capabilities are organizational, strategic routines and processes that enable the creation of new resource configurations in response to changing business environments and market structure [52]. Dynamic capability theory assumes that the major tools, which enables enterprises to achieve a competitive advantage, depends on the type of resources, and the way such resources are deployed [75]. According to the dynamic capability theory, competitive advantage might not necessarily originate from company resources, but it depends on how managers structure them. As a result, business owners/managers are key players in developing dynamic capabilities. Dynamic capabilities are strategic options that enable enterprises to (re)shape their existing resources in order to capitalize on opportunities in a volatile business environment [72].

Yet not all capabilities are dynamic [50]. To function as dynamic capabilities, resources must be capable of sensing, seizing, and transforming opportunities in the external business environment [87]. Business model innovation is the most common application of dynamic capabilities theory (BMI). Dynamic capabilities require perceiving, grabbing, and changing resources to enhance and direct current capabilities in order to innovate the existing business model, and as a result, create and reconfigure value delivered to customers [75, 92].

The business model is defined as the strategy used by businesses to create and deliver value, which contributes to business success [24, 35, 75, 89]. When enterprises recognized the need to rethink and reconfigure their strategy and value architecture in a turbulent business environment, the concept of business model innovation was introduced [40]. Business model innovation (BMI) is defined as the opportunity for existing organizations to renew their business models by altering or rethinking how they produce, offer, and acquire value through the different inter-related firm's core components [56]. According to the dynamic resource theory, BMI is the process of restructuring one or more inter-connected core elements that reinforce the process of creating and delivering value while allowing companies to detect and seize new possibilities to sustain or raise firm performance and competitiveness [22, 40].

Despite recent scholars' emphasis on the management of micro and small enterprises during crises, there is a dearth of research addressing the dynamic capabilities that act as drivers to BMI and improve organizational outcomes [40, 55]. There is a lack in empirical research on BMI in emerging economies in general as well as few attempts to pinpoint how dynamic capabilities explain business model innovation in micro and small enterprises. The majority of BMI studies have focused on large enterprises in developed countries. Nevertheless, as a practice-based discipline, the success of resilience strategies is influenced by context, which may limit the generalizability of these practices from developed to emerging economies [6]. Egypt is recognized as an emerging economy according to the International Monetary Fund organization.^1^ Though, emerging economies are not similar, they differ on the nature of business environment, country national strategies, and enterprise strategies [16]. Capabilities that are dynamic in one organization, in certain contexts, might fail to be dynamic in other contexts [69]. This highlights the need to investigate the business model innovation phenomenon and the driving dynamic capabilities at the level of micro and small enterprises in Egypt as an emerging economy. To stand on the dynamic capabilities boosting resilience strategies, it was necessary to scan the extant literature on resilience strategies of large firms, then reexamine their validity in the context of micro and small enterprises in an emerging economy.

Social capital is a well-known dynamic capability that received increased attention in the literature on large firms' resilience strategies following the COVID 19 crisis [4]. Social Capital Theory (SCT) is a useful theoretical lens for understanding how networks and relationships can enable resilience in turbulent business environment. Social capital has recently been acknowledged as a dynamic capability that supports the innovation capabilities of micro and small businesses with limited resources [64, 71]. In volatile business environments, managers can use their social capital to gain access to resources and information that would otherwise be unavailable [20]. Nonetheless, little is known about the relationship between social capital and the BMI of micro and small  enterprises  [71].

Market orientation (MO) is the second dynamic capability to reconsider in the context of micro and small enterprises. Adopting the right market orientation enable the detection and seizing of opportunities, as well as the making of timely and market-oriented decisions [25, 42–44, 82, 83, 86, 87]. The degree to which organizations adopt MO shapes their strategic flexibility and allows them to respond to changing levels and ranges of business environment uncertainty [27, 61, 86]. MO refers to the business philosophy and activities pursued by business managers or owners in order to collect data from clients and competitors and use pooled resources to generate value for clients as well as the entire company [23, 45]. Though MO is recognized indispensable for the resilience of firms in turbulent business environment, there is relative a scarcity of studies that investigated the role of market orientation as a dynamic capability in the context of micro and small enterprises [21].

This study contributes to the body of knowledge on business model innovation (BMI) in several aspects. First, it extends the concept of BMI, as a resilience strategy, to micro and small enterprises in an emerging economy. Second, it extends the concept of social capital as a dynamic capability from large size enterprises to micro and small ones, where it is relatively unresearched. Third, it bridges the gap between dynamic capabilities of micro and small enterprises and BMI. Fourth, this study is one of few that investigates the concept of market orientation in micro and small enterprises and examines its effect on both of BMI and business performance. Finally, this study provides a road map for micro and small enterprises on the drivers of business model innovation, as an affordable resilience strategy.

Building on the above theoretical foundations derived from extant literature, the following relationships discussed the antecedents and consequences of BMI in micro and small enterprises.

### BMI and business performance

Managers and researchers use the term "performance" to analyze the organization degree of success. Previous research employed both objective and subjective indices to measure business performance supported by the uncertainty of the business environment, the type of firm and the market condition. Objective performance metrics provide more insight into profitability and other financial indicators. Subjective metrics are related to market performance; some of which are non-financial and represent the organization's relationships with its customers, competitors, and suppliers [46]. Subjective performance metrics enable cross-industry comparison while supporting decision makers in identifying those aspects that are competent building facilitators and vital for an organization's strategic goals [79]. Non-financial measurements of performance may be more useful in measuring a company's competitive advantage regarding the increasing levels of environmental unpredictability [12, 28]. There is a consensus that large and small firms with innovative capabilities and innovative strategy can outperform their competitors in terms of financial and non-financial performance [13, 33]. Enterprises engaged in BMI was able to achieve competitive advantage and to sustainable growth, as recorded by [7, 18, 66]. Because of the informal nature of small businesses, financial performance measures are insufficient to capture company efforts to develop and survive [29, 65]. Based on the previous argument on BMI and business performance, the researchers propose the following hypotheses:

*H1. BMI positively affects Performance.*

*H1a. BMI positively affects financial performance.*

*H1b. BMI positively affects non-financial performance.*

### Managerial ties, BMI and Business Performance

Managerial ties are essential social capital qualities that should exist in top management networks. The importance of managerial ties to micro and small enterprises is originated from the crucial need for social capital to recognize and exploit opportunities [90]. It is recognized by managers and business owners as a strategic link to conserve resources, organize exchanges, overcome the constraints of existing infrastructure and, therefore, increase an organization's capacity for creativity and innovation [30, 59, 70, 76].

Scholars distinguish two forms of managerial ties: business ties and political ties [71]. Business relationships are horizontal connections between managers/owners and their customers, competitors, suppliers, and buyers that might be formal or informal. On the other hand, political ties are often informal, built on vertical interactions between government officials at various levels of administration [81]. Both forms of ties are vital for business survival [37]. Political ties are vital for large institutions because they allow them to access significant resources and gain strategic advantages, which can lead to greater company performance.

There is a consensus that networking serves as a blocking wall against uncalculated threats and risks from external business environment [14, 64]. However, this fact is contextual, the magnitude of the influence of managerial ties on business performance may vary with the strength of the institutions, the national political environment. In nations with low administrative corruption, local political links boost commercial strategy and, eventually, corporate performance [5, 74, 76]. However, in others suffering high corruption, a study reported a negative relationship between the regulatory environment and corporate performance [2]. Moreover, it is argued that political ties can boost business performance, especially if the management's reputation is good or the company's prior performance is better than its competitors [57, 67]. In some contexts, protective relations, with political linkages, are more advantageous to weaker enterprises [80, 90].

In some economies, business ties are more profitable when legal procedures are ineffective and technology advances rapidly, but political ties improve performance when government support is weak, and technical developments are delayed [73, 90].

To sum up, the need for managerial ties is substantial to micro and small enterprises in emerging markets which face ill-business regulations and scattered assistance. Accordingly, the following hypotheses are proposed:

*H2. Managerial ties positively affect business performance.*

*H2a: Business ties positively affect financial performance.*

*H2b: Business ties positively affect non-financial performance.*

*H2c: Political ties positively affect financial performance.*

*H2d: Political ties positively affect non-financial performance*

*H3. Managerial ties positively affect BMI.*

*H3a. Business ties positively affect BMI.*

*H3b. Political ties positively affect BMI.*

### Environmental uncertainty and BMI

Environmental uncertainty is defined as rapid shifts in consumer demand and instability in the company external environment. Environmental Uncertainty results in the decision-makers’ lack of knowledge about environmental conditions, limiting their ability to anticipate the future [10, 18, 46]. Given that the levels of uncertainty in business environment might differ among countries, the perception of uncertainty levels is what matters for decision makers and entrepreneurs [9].

Despite that environmental uncertainty can have a negative impact on a company's performance, experts have identified the importance of environmental uncertainty to innovation in both large and small businesses. It is argued that in low dynamic environments, organizations’ dynamic capabilities are underutilized [72]. On the other hand, it is believed that high level of uncertainty encourages creativity and compels firms to challenge the status quo, increase their resource integration capacity and accelerate the innovation process [26]. Hence, the following hypothesis can be developed:

*H4: The higher the perceived business environment uncertainty, the higher levels of innovation in Business Model.*

### Perceived environmental uncertainty and MO

Business Environmental uncertainty is one of the main drivers to adopt market orientation [80, 82]. To survive in a high level of business environment uncertainty, higher level of MO is required as it enables the organization or its owner market learning and flexibility [27, 80, 84]. MO decides how organizations experience turbulent business environment behave to sustain or improve competitive environment.

Unlike large enterprises, where MO process is more formalized and complex, in micro and small enterprises, where resources are limited, and decisions are mostly centralized in the hands of their owners. Hence, in small and micro enterprises MO depends on the competencies and behavioral characteristics of the owners/managers [36]. Because of the informality and personal relationships, they have with stakeholders, micro and small business owners are seen as capable of practicing MO, allowing them to maneuver and seize market opportunities while adapting to business environment uncertainties [27, 68, 78].

Reactive and proactive MOs are two pillars yet not contradictory. They figure the strategic flexibility, shape the value creation strategy, influence the level and scope of business networking (ties), enable organizations to utilize their capabilities and respond to changing business environments [15, 85]. Reactive MO refers to a company's ability or its owner to react to environmental changes; whereas proactive MO refers to the ability of the company or business owner to model, shape or transform the business environment [56, 84]. Reactive MO is market-driven which accepts market changes as given and displays more exploitative behavior through focusing on customers' existing needs and, accordingly, their reactions. As for proactive MO, it is market-driving which focuses on customers' latent and future needs through exhibiting more explorative behavior and developing new knowledge about the firm's markets, products, and capabilities [49, 68]. Building on previous discussion, the researchers propose that:

*H5: Higher levels of perceived environmental uncertainty triggers higher levels of market orientation.*

*H5a: Higher levels of perceived environmental uncertainty triggers higher levels of proactive market orientation.*

*H5b: Higher levels of perceived environmental uncertainty triggers higher levels of reactive market orientation.*

### Market orientation and BMI

Previous researchers confirmed a relation among intensity, types of market orientation and degrees of innovation [40, 49, 50, 54, 61, 78]. Both orientations are core requirements for competitive advantage and have proved a positive impact on innovation and BMI [30, 78, 80, 86, 89], Based on the above theoretical arguments, the researchers propose the following hypotheses:

*H6: Market orientation positively affects BMI.*

*H6a: Proactive MO positively affects BMI.*

*H6b: Reactive MO positively affects BMI.*

### Market orientation and business performance

There is a consensus that practicing MO nurtures higher levels of customer loyalty and superior customer value; all of which resulted in higher organizational performance [11, 47, 85, 86].

*H7: MO positively affects business Performance.*

*H7a: Proactive MO positively affects non-financial performance.*

*H7b: Proactive MO positively affects financial performance.*

*H7c: Reactive Market orientation positively affects non-financial performance.*

*H7d: Reactive Market orientation positively affects financial performance.*

### The mediating effect of BMI

The impact of management ties, on business ability to innovate, is as significant as its impact on business performance. Managerial relationships can enable businesses to innovate. It was found that managerial ties, with government agencies and universities in Malaysia as an emerging economy, enhance inward and outbound open innovation in high-tech industries [58]. It is confirmed that management links have a direct and indirect impacts on BMI through improving opportunity recognition and exploratory learning in a study to evaluate the impact of managerial ties on BMI [81]. According to several scholars, both political and business links help businesses to access: dynamic external resources, up-to-date knowledge, legal protection, and cost savings besides positively enhancing innovative ability and performance [8, 11, 34, 58, 80, 89]. Managerial ties enable businesses to reap the rewards of these connections. They provide enterprises with resources needed to boost innovations, hence, improving business performance and sustaining their competitive edge [8, 11, 70]. Accordingly, the researchers propose the following hypotheses:

*H8. BMI positively mediates the relationship between managerial ties and business performance.*

*H8a: BMI positively mediates the relationship between managerial ties and business financial performance.*

*H8b: BMI positively mediates the relationship between managerial ties and business non-financial performance.*

The effect of MO on organizational performance was found to be partially or fully mediated by innovation practices and products [31]. Most of studies addressed the effect of MO on business performance and innovative ability were initiated in developed countries where large or SMEs are seen as market oriented [21]. This calls for re-examining these relationships in an emerging economy, in which the micro and small enterprises are in need for innovation to survive in a highly competitive and uncertain business environment. Hence, researchers can propose the following hypotheses:

*H9: BMI mediates the relationship between marketing orientation and business performance.*

*H9a: BMI mediates the relationship between reactive MO and business performance.*

*H9b: BMI mediates the relationship between proactive MO and business performance.*

## Methodology

### Research instrument

**Fig. 1:**
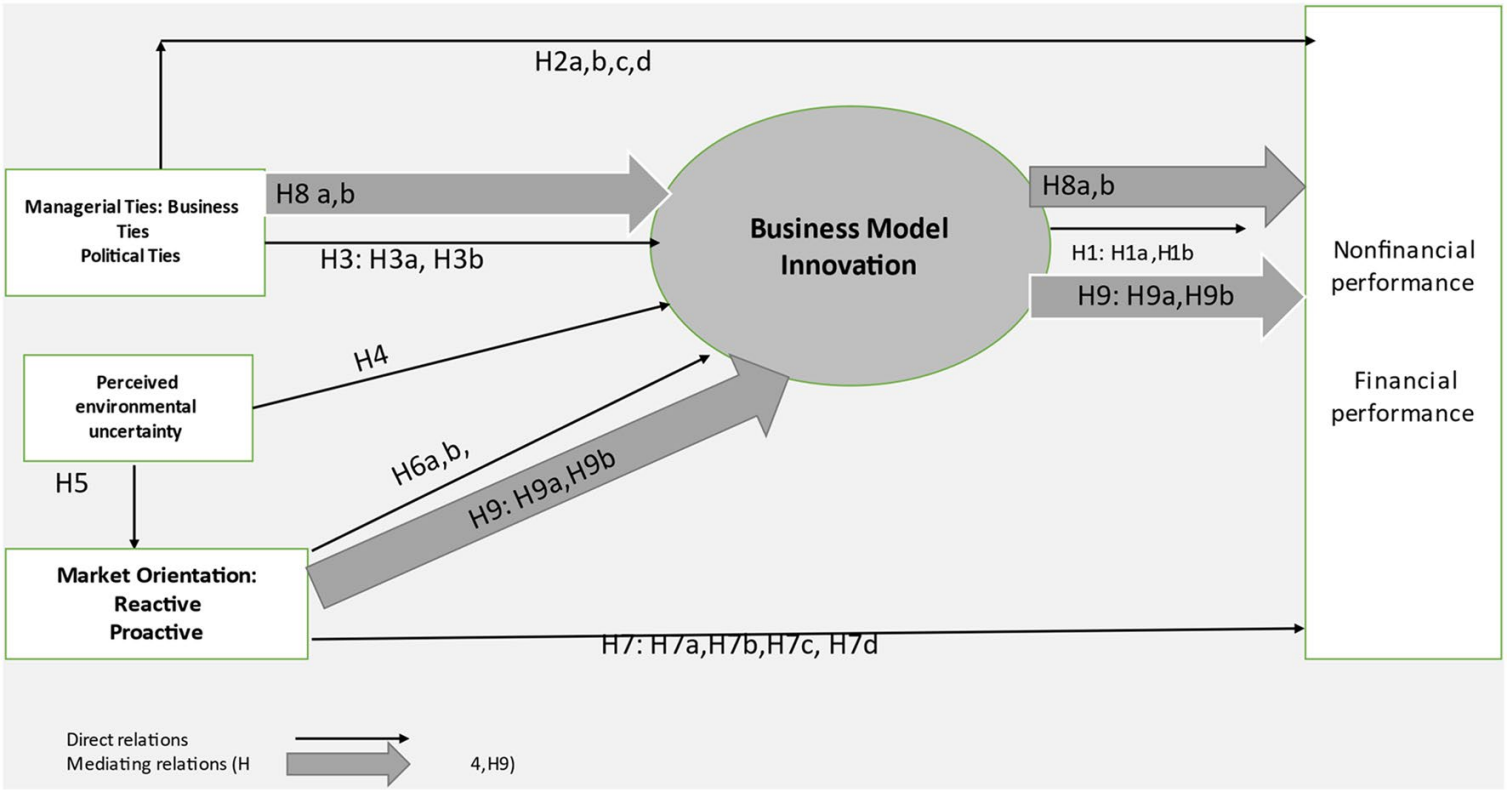
Research model. *Source*: the researchers

This research employed a quantitative approach, through surveying micro and small enterprises. The researchers used the questionnaire as the main tool to collect the data needed to analyze the relationships in the research model, Fig. 1. Face-to-face structured interviews were used to collect data from sample units that agreed to participate in the study. This was accomplished with the help of a paid team of young researchers.

The responses were measured using a five-point Likert scale (5 Strongly Agree and 1 Strongly Disagree). Scales measuring variables, are adopted from literature and adapted to suit Arabic language speakers. Managerial ties, including business and political ties is adopted from [70]; perceived environmental uncertainty is adopted from [88]; Business model innovation is adopted from [22]. Business performance, with its two dimensions: financial and non-financial aspects, is derived from [46]. Finally, proactive and reactive MO was adopted from [86].

### Analysis

This study used partial least squares based-structural equation modeling method PLS 3.3 to analyze data output from the survey in order to overcome the problems related to normality [38]. The measurement model was tested to ensure its reliability and validity. Multiple indicators assure the discriminant and convergent validity for the model variables. Cross loadings, AVE and Fornell-Larcker criterion were used to assess discriminant validity. Also, the variance inflation factor (VIF), was used to evaluate collinearity of the formative indicators.

### Sampling technique

Although the Central Agency for Public Mobilization and Statistics (CAPMAS) reported that the number of micro and small enterprises exceeded three million in 2020, it was difficult to find a consolidated, up to date, sampling frame that includes the names, industry sector, and addresses of the micro and small enterprises. As a result, we had to construct a research sample frame from which the sample was drawn. We based our population of micro and small businesses on the Ministry of Industry's directory of Egyptian industries, which includes 30 industry sectors. Four criteria have been used to build the sampling frame. The first criterion is the amount of capital required to start a business in the industry sector, which qualifies some industries for exclusion. The second criterion is the proximity to Greater Cairo. The third criterion is the business owner acceptance to participate in this study. While the fourth criterion is to ensure that the participating enterprises capital qualify them to be  classified as micro and small enterprises according to law no. 152 for 2020 for SMEs.

Applying the first three criteria, we ended with a pool of small and micro enterprises representing selected industries operating in greater Cairo. They are detergents and disinfectants industry, glass industry, leather industry and tanneries industry, wooden and furniture industry, textile and readymade garments industry, packing and packaging industry, plastic industry, and electrical and electronics industry. The enterprises in these sectors were approached by the data collection team, to filter them on willingness to participate in the study. To ensure that the participating enterprises are micro or small, screening questions on capital was added to the questionnaire before approaching the sample units.

To sum up, although nonprobability judgmental sampling technique was used to select sample units, it was based on logical and justified criteria to ensure minimum selection bias and maximum participation from the selected enterprises.

## Results

### Sample profile

In total, 426 survey responses were used in the analysis collected from micro and small enterprises in order to test the pre-determined hypotheses. Results show that 85% of the participating enterprises employ less than 10 employees. The majority which represents 55% of the participating enterprises capital is less than 500 thousand Egyptian pounds (25 thousand dollars^2^), while 30% of the participating enterprises capital is larger than 500 thousand and less than one million Egyptian pounds (50 thousand dollars), with the remaining 15% of participating enterprises capital does not exceed 2 million Egyptian pounds. This qualifies the participating enterprises to be categorized as a micro and a small enterprise according to law no. 152, for 2020, that was launched by the Egyptian government to recognize and differentiate among micro, small and medium enterprises.

Concerning the legal form of the responding businesses, the study descriptive statistics show that 56% of the sample are sole proprietorship while 34% of the sample are partnership-based enterprises. Besides, results show that 77% of the sample depends on the business owner own funds as the main source of capital. Furthermore, 53% of the participating enterprises are offering standardized products while 47% are rendering customized products. The data shows that 20% only of the sample owns patent, and results show that the 90% of the sample serves domestic market while 10% serves international markets.

### The measurement model

The internal reliability and validity of the measurement model shows satisfactory measures as shown in Table 1. Values of Cronbach alpha are all 0.7 at minimum as recommended by [38]. Moreover, the values of composite reliability (CR) are all more than 0.8 which is above the threshold as advised by [41]. The rho_A < 1 for all latent variables. Table 1 shows that AVE values are all greater than 0.5. Fornell-Larchker is a criterion for assessing discriminant validity that compares the square root of the AVE values with the latent variable correlations. As shown in Table 1, the  square roots of the AVE, for each construct (proving the convergent validity) shown by the diagonal italic figures, are greater than their highest correlation value with any other construct; this  agrees with Fornell-Larchker criterion. Table 2 shows loadings are ≥ 0.7 approximately, with no cross loadings shown. The VIF shows that all measures do not exceed five, the cut-off as recommended by [27, 38, 41]. As for Heterotrait-monotrait (HTMT) ratio of correlation, it recorded less than 0.9. Thus, convergent and discriminant validity of the measurement model are satisfied.

**Table 1:** Latent variables correlation, reliability and validity

	BMI	Political ties	Business ties	Financial performance	Non-financial performance	Perceived uncertainty	Proactive	Reactive
BMI	*0.774*							
Political TIES	0.196	*0.816*						
Business ties	0.440	0.223	*0.917*					
Financial performance	0.244	0.278	0.158	*0.876*				
Non-fin performance	0.400	0.313	0.214	0.602	*0.778*			
Perceived environmental uncertainty	0.272	0.095	0.071	0.086	0.151	*0.822*		
Proactive MO	0.487	0.207	0.339	0.273	0.403	0.275	*0.792*	
Reactive MO	0.525	0.158	0.343	0.232	0.316	0.142	0.449	*0.754*
Cronbach's Alpha ≥ 0.7	0.833	0.749	0.906	0.898	0.837	0.762	0.703	0.751
rho_A < 1	0.839	0.752	0.907	0.900	0.842	0.835	0.704	0.763
Composite Reliability ≥ 0.8	0.882	0.856	0.941	0.929	0.884	0.861	0.835	0.840
Average Variance Extracted (AVE) ≥ 0.5	0.599	0.666	0.842	0.767	0.605	0.675	0.627	0.568

**Table 2:** Variables loadings, VIF

	BMI	Political ties	Business ties	Financial performance	Non-fin performance	Perceived environmental uncertainty (PEU)	Proactive	Reactive	VIF
BMI1	0.778								1.820
BMI2	0.787								1.862
BMI3	0.798								1.919
BMI4	0.787								1.852
BMI5	0.715								1.621
BT1			0.909						2.793
BT2			0.925						3.229
BT4			0.918						2.888
FP1				0.876					3.185
FP2				0.904					3.547
FP3				0.822					1.918
FP4				0.898					2.843
MOPRO2							0.797		1.355
MOPRO6							0.777		1.344
MOPRO7							0.801		1.426
MOR1								0.791	1.443
MOR3								0.775	1.604
MOR4								0.697	1.459
MOR5								0.749	1.320
NFP1					0.796				1.793
NFP2					0.760				1.645
NFP3					0.802				1.899
NFP4					0.800				1.850
NFP5					0.727				1.682
PEU1						0.906			1.823
PEU2						0.804			1.668
PEU3						0.748			1.380
PT1		0.802							1.417
PT2		0.820							1.624
PT3		0.825							1.514

### Structural model

**Fig. 2:**
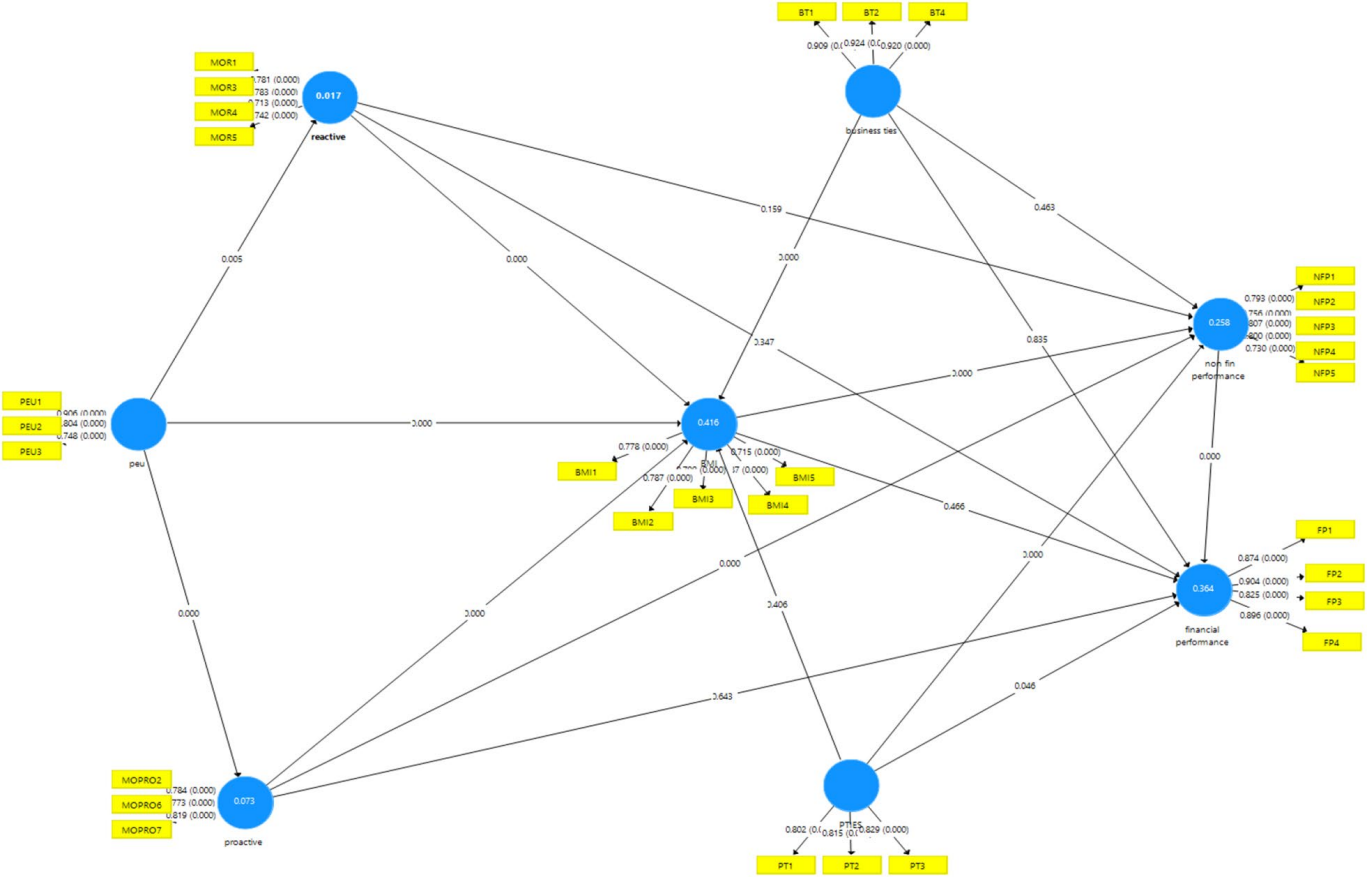
Model tested*.* Source*: Smart PLS 3.3 output*

The significance and direction of the hypothesized relationships were tested using the bootstrap method with 5000 subsamples. PLS-SEM indices, for model fit, involved SRMR = 0.101 ≤ 0.12 as recommended [38, 48], NFI = 0.777, and d_G = 0.574. Figure 2 and Table 3 show that the perceived environmental uncertainty, significantly, affects both of reactive and proactive marketing orientation with (β = 0.139, β = 0.274), respectively. In addition, Table 3 shows that business ties (β = 0.242); perceived environmental uncertainty (β = 0.149); proactive marketing orientation (β = 0.215) and reactive marketing orientation (β = 0.317) are significantly responsible for 41.6% in the BMI. Moreover, Fig. 1 shows that adjusted R square, for non-financial performance, is 25.8% significantly affected by BMI (β = 0.222) with political ties (β = 0.219) and proactive orientation (β = 0.23) and p ≤ 0.001. Finally, both Table 3 and Fig. 2 prove that 36.4% of the financial performance is explained by the non-financial performance with β = 0.559, t = 14.671, p ≤ 0.001 and political ties with β = 0.096 and p ≤ 0.05. Table 3 displays the results of testing direct hypotheses, supported by *P* values indicating significance levels.

**Table 3:** Direct relationships (All hypotheses except H9, H8)

	Hypotheses		Original sample (O)	Sample mean (M)	Standard deviation (STDEV)	T statistics (|O/STDEV|)	*P* values	Accept/reject
H1	H1a	BMI—> financial performance	− 0.039	− 0.040	0.053	0.730	0.466	Reject
	H1b	BMI—> non-fin performance	0.222	0.220	0.057	3.864	0.000	Accept
H2	H2a	business ties—> financial performance	0.010	0.010	0.050	0.208	0.835	Reject
	H2b	business ties—> non-fin performance	− 0.036	− 0.036	0.050	0.733	0.463	Reject
	H2c	PTIES—> financial performance	0.096	0.097	0.048	1.995	0.046	Accept
	H2d	PTIES—> non-fin performance	0.219	0.221	0.048	4.536	0.000	Accept
H3	H3b	PTIES—> BMI	0.034	0.035	0.041	0.831	0.406	Reject
	H3a	business ties—> BMI	0.242	0.242	0.047	5.096	0.000	Accept
H4	H4	PEU—> BMI	0.149	0.149	0.042	3.513	0.000	Accept
H5	H5a	PEU—> Proactive	0.274	0.277	0.047	5.854	0.000	Accept
	H5b	PEU- > Reactive	0.139	0.141	0.049	2.817	0.005	Accept
H6	H6a	proactive—> BMI	0.215	0.216	0.051	4.240	0.000	Accept
	H6b	reactive—> BMI	0.317	0.317	0.046	6.892	0.000	Accept
H7	H7a	proactive—> non-fin performance	0.230	0.233	0.059	3.916	0.000	Accept
	H7b	proactive—> financial performance	0.024	0.025	0.052	0.463	0.643	Reject
	H7c	reactive—> non-fin performance	0.076	0.077	0.054	1.408	0.159	Reject
	H7d	reactive—> financial performance	0.048	0.048	0.051	0.940	0.347	Reject

### Direct relations

Table 3 confirms that BMI partially affects non-financial performance, while failed to directly affect financial performance. Hence H1 is partially accepted. Moreover, as Table 3 shows a significant direct effect from political ties on both financial and non-financial performance. However, business ties failed to show positive direct effect on both financial and non-financial performance. Thereby, H2 is partially accepted. Table 3 confirms that business ties, not political ties, boosts BMI, providing evidence to partially accept H3. In addition, H5 is accepted, however, the effect of PEU on proactive marketing orientation is higher than its effect on reactive marketing orientation. Moreover, Table 3 confirms that both reactive and proactive MO are affecting BMI positively, thereby H6 is accepted. Finally, Table 3 confirms the partial acceptance of H7.

## Testing mediation relationships

To test the mediation analysis, bootstrapping, with 5000, was conducted as recommended [38]. Tables 4 and 5 are used in the mediation analysis.

**Table 4:** Indirect relationships

	Original sample (O)	Sample mean (M)	Standard deviation (STDEV)	T statistics (|O/STDEV|)	*P* values
BMI—> financial performance	0.124	0.123	0.034	3.688	*0.000*
PTIES—> financial performance	0.125	0.127	0.029	4.345	*0.000*
PTIES—> non-financial performance	0.008	0.008	0.010	0.782	*0.434*
Business ties—> financial performance	0.000	0.000	0.030	0.009	*0.993*
Business ties—> non- financial performance	0.054	0.053	0.017	3.138	*0.002*
Proactive—> financial performance	0.147	0.148	0.035	4.243	*0.000*
Proactive—> non- financial performance	0.048	0.047	0.016	2.936	*0.003*
Reactive—> financial performance	0.069	0.069	0.035	1.966	*0.049*
Reactive—> non- financial performance	0.070	0.070	0.021	3.303	*0.001*

**Table 5:** Specific indirect effect

		Original sample (O)	Sample mean (M)	Standard deviation (STDEV)	T statistics (|O/STDEV|)	*P* values	Mediation/No mediation
H8	PTIES—> BMI—> financial performance	− 0.001	− 0.002	0.003	0.387	0.699	No mediation
	Business ties—> BMI—> financial performance	− 0.009	− 0.010	0.013	0.705	0.481	No mediation
	PTIES—> BMI—> non-fin performance	0.008	0.008	0.010	0.782	0.434	No mediation
	Business ties—> BMI—> non-fin performance	0.054	0.053	0.017	3.138	0.002	Mediation
H9	Proactive—> BMI—> financial performance	− 0.008	− 0.009	0.012	0.691	0.490	No mediation
	Reactive—> BMI—> financial performance	− 0.012	− 0.013	0.017	0.722	0.471	No mediation
	Proactive—> BMI—> non-fin performance	0.048	0.047	0.016	2.936	0.003	Mediation
	Reactive—> BMI—> non-fin performance	0.070	0.070	0.021	3.303	0.001	Mediation

Table 4 shows significant indirect relationship between BMI and perceived environmental uncertainty PEU (β = 0.13, *t* = 4.485). In addition, results show a significant indirect relationship between BMI and financial performance with β = 0.124 and *t* = 3.688. Moreover, a significant indirect relationship exists between political ties and financial performance (β = 0.125, *t* = 4.345). Furthermore, Table 4 displays a significant indirect relationship between business ties and non-financial performance (β = 0.054, *t* = 3.138). In addition, it confirms a significant indirect relationship between proactive MO and each of financial performance and non-financial performance with β = 0.147 and β = 0.048, respectively. Moreover, Table 4 shows a significant existing indirect relationship between reactive MO and each of the financial and non-financial performance with β = 0.069 and β = 0.070, respectively. *P* values indicating significance levels are depicted in Table 4.

While Table 4 shows the significant indirect effect, Table 5 clarifies the contribution of the mediator to the indirect effect. Hence, the mediation hypotheses are accepted or rejected.

Table 4 shows a significant indirect relationship between business ties and non-financial performance. As shown in Table 5, it ensures the mediation of BMI in the relationship between business ties and non-financial performance with VAF = 100%, indicating a full mediation [62]. However, Tables 3 and 4 ensure a significant indirect relationship between political ties and financial performance through non-financial performance not through the BMI. Thus, H8 is partially accepted.

As shown in Table 5, BMI does not mediate the relationship between each of reactive MO and proactive MO with financial performance while BMI mediates the relationship between reactive MO and non-financial performance with VAF = 48.6%, this signals partial mediation. In addition, Table 5 reports a significant mediation of BMI in the relationship between proactive MO and business performance with VAF = 17.2% < 20%; this indicates poor mediation. Therefore, H9 is partially accepted. Moreover, Table 5 shows a significant indirect relationship between BMI and financial performance; Table 4 ensures that non- financial performance mediates the relationship between BMI and financial performance (β = 0.124, *t*- = 3.688) with VAF = 100%; indicating a full mediation.

## Conclusion

This study was initiated to identify the drivers and consequences of business model innovation in micro and small enterprises. This study investigates the impact of managerial ties, business owner market orientation and perceived environmental uncertainty as drivers to BMI. Also, it investigates the effect of BMI on business performance. According to the findings, the adjusted R square is 41.6% for BMI, 36.4% for financial performance, and 25.8% for non- financial performance. All are considered of moderate rather than strong explanatory power [38]. Also, Fig. 2 shows adjusted R square of 1.7% for reactive MO, and 7.3% for proactive MO, considered as of poor explanatory power. Results show that not all assumed capabilities derived from literature, are considered as dynamic for micro and small enterprises in the Egyptian economy.

Like previous studies, the results of this study confirm that business ties, a type of management ties, is one of the key dynamic capabilities and drivers of BMI. However, the findings of this paper confirm that political ties did not, significantly, contribute to building BMI of micro and small business in the Egyptian economy, which contradicts with studies on micro and small enterprises in other emerging economies [8, 34]. Unlike large corporations, micro and small enterprises, in some emerging countries especially Egypt, may not be able to build political ties that can significantly enable BMI. Consistent with previous studies, it can be concluded that not all managerial ties are enablers to innovation and /or BMI [70, 81]. This could be due to the low level of trust that exists between political parties and businesses in general and in Egypt in particular [44]. When compared to large corporations, micro and small enterprises may have less influence on political parties in some emerging countries. Other probable explanation holds that this influence depends on how political ties are relationally, structurally, and cognitively configured [77]. Business bargaining power over political ties depends to a great extent, on business strength in the market; this is, in addition to other contextual market considerations [34, 91].

Likewise, previous scholars confirm the importance of an uncertain business environment in BMI as an innovation strategy [3, 46]. Similarly, our study results show that H4 is accepted, implying that the more a business owner is uncertain about the external environment and feels threatened, the more inclined they are to modify the status quo and think creatively. However, this result must be taken with cautions as high levels of uncertainty are accompanied with the absence of dynamic capabilities might obstruct innovation.

Our findings confirm that H6 is accepted. This goes in line with previous scholars [17, 31, 55, 87], emphasizing the importance of both proactive and reactive marketing orientations in driving BMI and contributing positively to business performance. However, the strength of the effect of proactive MO and reactive MO on BMI is not equal. It has been argued that proactive MO is more explorative in sensing external environment through allowing small business owners/managers to capitalize dynamic capabilities, build potential networks, develop new markets and triggers innovation than reactive orientation [39, 60, 63, 68]. Surprisingly, the findings of this paper show that reactive MO has a more influence on BMI and non-financial performance than proactive marketing orientation. This means that for owners of micro and small enterprises with limited resources, it is easier to adopt a reactive MO through exploitative seizing of opportunities and reconfiguring existing resources to implement their BMI [68, 87]. On the other hand, adopting proactive MO requires intensive resources and long-term planning [82]. This might be inaccessible with the unstable nature of the Egyptian economy, that may restrict entrepreneurs' capacity to use exploratory sensing techniques in formulating their business plan. Another plausible reason, for the higher effect of reactive marketing orientation on BMI, is that micro and small business owners may be reactive-oriented at the starting phase while they tend to be proactive as their businesses grow and gain market knowledge [36]. In addition, adopting proactive orientation is risky and derived by highly perceived level of environmental uncertainty as shown in the results and confirmed by H5. Moreover, adopting proactive marketing orientation is derived by high level of entrepreneurial orientation which depends, to a large extent, on the prevailing institutional environment [1].

Our study findings confirmed by H1 ensures that BMI directly improves non-financial business performance, thereby is consistent with earlier research [51, 74]. Yet, BMI indirectly improves non-financial performance. This implies that owners of micro and small enterprises should expect a gap between both types of performance.

## Implications

With the difficulties encountered in researching micro and small enterprises, this sector has been overlooked by scholars, especially in emerging economies. This study is one of the few studies that address the business model innovation in micro and small enterprises in Egypt as an emerging economy. On the academic level, this research contributes to the business model innovation body of knowledge by identifying those dynamic capabilities contributing to BMI of micro and small enterprises and contribute to improve their performance in a highly dynamic business environment. This study confirms that dynamic capabilities in micro and small enterprises are more than tangible resources; they are a mix of intangible resources which mostly depends on the owner\ founder skills and capabilities. Moreover, this study ensures that social capital especially business ties, are one of the pillars of BMI while political ties failed to affect BMI, unlike results in other developed and emerging countries. This implies that it might be difficult to generalize the findings of micro and small business from one emerging economy to the other. Researchers should be cautious when generalizing results along emerging economies on the micro and small business sector. Furthermore, this research is one of few that recognizes the role of micro and small business owners marketing orientation in driving BMI.

From a managerial standpoint, the results re-assure that BMI is a strategy by which micro and small business can adapt to environmental uncertainty. Thereby, micro, and small business owners must acknowledge that innovation is not only in product but also inherited in the configuration of dynamic tangible and intangible capabilities. The study results imply that micro and small enterprises encounter difficulties with fostering political ties. This highlights the need of policymakers in the governmental bodies to establish several communication channels with micro and small business owners and ease the difficulties they encounter. Government bodies can contribute to reduce the cost and difficulties of establishing managerial ties: business and political. This can be accomplished by offering free information about competitors, markets, as well as potential suppliers and areas of potential investment opportunities. Moreover, the government should expand micro and small business clusters through gathering similar industries to foster cooperation, knowledge sharing in order to reduce the cost of developing those business [39].

Furthermore, this study results assure that the role of micro and small enterprises owners/managers, is significant in resource deployment. This study, unlike others, has acknowledged the need for both proactive and reactive MO of business owner/manager in driving innovation. Our findings assert that reactive marketing orientation is as important as proactive marketing orientation in addressing environmental uncertainty and pursuing innovation. This highlights the role of training and educating micro and small business owners on how and when to adopt reactive and proactive marketing orientation.

Finally, our study findings ensure that performance of micro and small enterprises should not be assessed only by financial performance, but also it should incorporate non-financial performance measures, as well. Micro and small business owners/managers should acknowledge the fact that in order to improve financial performance, improving non-financial performance is a predecessor.

## Limitations and further research directions

This study, like other studies, has limitations that should be considered in future studies. This study's sample is restricted to micro and small businesses. Incorporating large corporations adds more insights into the drivers of business model innovation and provides a thorough understanding of the contexts in which such drivers act as dynamic capabilities. While this study investigates the role of managerial ties as an organizational capability, it does not investigate the embeddedness of such relationships in depth. This aspect needs to be further investigated to provide insights into how to strengthen such ties. Moreover, other dynamic capabilities can be considered in future research. Despite the role of uncertain business environment in articulating micro and small business country level strategies, this study did not address the contextual factors that contribute to perceived environmental uncertainty in detail; therefore, it should be the focus of future research. Future research may explore the barriers of business model innovation among micro, small and medium enterprises. Moreover, future research can replicate this study with more focus on the comparison among BMI drivers along the industry sectors. Finally, future research can differentiate between dynamic and non-dynamic capabilities of micro and small enterprises in different industry sectors.

## Appendix

See Table

**Table 6:** Scales measuring Study model variables and their sources

Managerial ties:	Political ties:
Source: Sami, P., Rahnavard, F., & Tabar, A. A. (2019). The effect of political and business ties on firm performance: The mediating role of product innovation. *Management Research Review*. Vol. 42 No. 7, 2019 pp. 778–796	1. My company has good relations with officials at various levels of government
	2. My company has good relations with government officials in regulatory organizations
	3. My company relationships with government officials at the provincial level have been good
	4. My company spends considerable time and energy to establish effective communication with government officials
	Business Ties
	1. My company has good relations with their counterparts in supplier firms
	2. My company has good relations with their counterparts in buyer companies
	3. My company has good relations with their counterparts in competing firms
	4. My company has good relations with their counterparts in marketing-based collaborators
	5. My company has good relations with their counterparts in technological collaborators
	6. My company has good relations with universities to conduct R&D activities
*Market orientations*	*Reactive:*
Source: Yang, D., Wei, Z., Shi, H., & Zhao, J. (2020). Market orientation, strategic flexibility and business model innovation. Journal of Business & Industrial Marketing. 35/4 (2020) 771–784)	1. We measure customer satisfaction systematically and frequently
	2. We are more customer-focused than our competitors
	3. We freely communicate information about our successful and unsuccessful customer experiences across all business functions
	4. Data on customer satisfaction are disseminated at all levels in this company on a regular basis
	5. Our strategy for competitive advantage is based on our understanding of needs
	*Proactive:*
	1. We continuously try to discover additional needs of our customers of which they are unaware
	2. We continuously try to discover additional needs of our customers of which they are unaware
	3. We extrapolate key trends to gain insight into what customers will need in the future
	4. We incorporate solutions to unarticulated customer needs in our products and services
	5. We brainstorm on how customers use our products and services
	6. We innovate even at the risk of making our own offering obsolete
	7. We work with lead users of our offerings to recognize customer’ needs months in advance of the majority of users
	8. We search for opportunities in areas where customers have a difficult time expressing their needs
*Perceived environmental uncertainty*	1. The needs and wants of our customers are changing very fast
Source: Zhang, D., Linderman, K., & Schroeder, R. G. (2012). The moderating role of contextual factors on quality management practices. *Journal of Operations Management*, *30*(1–2), 12–23	2. The demand for our plant’s products is unstable and unpredictable
	3. Our competitive pressures are extremely high
*Mediator: Business model innovation*	1. When necessary, we are able to carry out massive internal reconfigurations to enhance our overall value proposition to our customers
Source: Ciampi, F., Demi, S., Magrini, A., Marzi, G., & Papa, A. (2021). Exploring the impact of big data analytics capabilities on business model innovation: The mediating role of entrepreneurial orientation. Journal of Business Research, 123, 1–13	2. When we sense an opportunity, we are quick at re-organizing our operating processes
	3. When necessary, we are able to reorganize our partner network to improve our value proposition to our customers
	4. New opportunities to serve our customers are quickly understood
	5. We regularly consider innovative opportunities for changing our existing pricing models
*Dependent: Performance*	
Source: Kafetzopoulos, D., Psomas, E., & Skalkos, D. (2019). Innovation dimensions and business performance under environmental uncertainty. European Journal of Innovation Management. Vol. 23 No. 5, 2020 pp. 856–876	*Financial aspects of performance*:
	1. Gross margin has improved
	2. Profit levels have improved
	3. Productivity has improved
	4. Return on investment has improved
	*Non -Financial aspects of performance:*
	5. Customer satisfaction has improved
	6. Product quality has improved
	7. The knowledge on how to efficiently manage operations has increased
	8. Business relationships with partners, distributors and suppliers have improved
	9. Employee retention has improved

6.

## Abbreviations

SMEs: Small and medium enterprises.
BMI: Business Model Innovation.
MO: Market orientation

## Footnotes

1 https://www.imf.org/en/News/Articles/2021/07/14/na070621-egypt-overcoming-the-covid-shock-and-maintaining-growth#:~:text=Egypt%20was%20one%20of%20the%20few%20emerging%20market%20countries%20that,the%20face%20of%20the%20pandemic.

2 Equivalency in dollars is calculated based on the exchange rate announced by the central bank of Egypt on September 11, 2022.

## References

[1] Adel HM, Mahrous AA, Hammad R (2020). Entrepreneurial marketing strategy, institutional environment, and business performance of SMEs in Egypt. J Entrep Emerg Econ.

[2] Adomako S, Danso A (2014). Regulatory environment, environmental dynamism, political ties, and performance: Study of entrepreneurial firms in a developing economy. J Small Bus Enterp Dev.

[3] Afshar Jahanshahi A, Brem A (2020). Entrepreneurs in post-sanctions Iran: Innovation or imitation under conditions of perceived environmental uncertainty?. Asia Pacific J Manag.

[4] Al-Omoush KS, Ribeiro-Navarrete S, Lassala C, Skare M (2022). Networking and knowledge creation: Social capital and collaborative innovation in responding to the COVID-19 crisis. J Innov Knowl.

[5] Amore MD, Bennedsen M (2013). The value of local political connections in a low-corruption environment. J Financ Econ.

[6] Anderson A, Ronteau S (2017). Towards an entrepreneurial theory of practice; emerging ideas for emerging economies. J Entrep Emerg Econ.

[7] Anwar M (2018). Business model innovation and SMEs performance—does competitive advantage mediate?. Int J Innov Manag.

[8] Anwar M, Shah SZ (2021). Entrepreneurial orientation and generic competitive strategies for emerging SMEs: financial and nonfinancial performance perspective. J Public Affairs.

[9] Arend RJ (2022). How uncertainty levels and types matter, to likely entrepreneurs and others. J Business Venturing Insights.

[10] Astuty W, Pasaribu F, Rahayu S, Habibie A (2021). The influence of environmental uncertainty, organizational structure and distribution network competence on the quality of supply chain management information systems. Uncertain Supp Chain Manag.

[11] Bamfo BA, Kraa JJ (2019). Market orientation and performance of small and medium enterprises in Ghana: the mediating role of innovation. Cogent Business Manag.

[12] Bastian E, Muchlish M (2012). Perceived environment uncertainty, business strategy, performance measurement systems and organizational performance. Procedia Soc Behav Sci.

[13] Bayraktar CA, Hancerliogullari G, Cetinguc B, Calisir F (2017). Competitive strategies, innovation, and firm performance: An empirical study in a developing economy environment. Technol Anal Strategic Manag.

[14] Boso N, Story VM, Cadogan JW (2013). Entrepreneurial orientation, market orientation, network ties, and performance: Study of entrepreneurial firms in a developing economy. J Bus Ventur.

[15] Brozovic D (2018). Strategic flexibility: A review of the literature. Int J Manag Rev.

[16] Bruton GD, Filatotchev I, Si S, Wright M (2013). Entrepreneurship and strategy in emerging economies. Strateg Entrep J.

[17] Bucktowar R, Kocak A, Padachi K (2015). Entrepreneurial orientation, market orientation and networking: impact on innovation and firm performance. J Dev Entrep.

[18] Buliga O, Scheiner CW, Voigt KI (2016). Business model innovation and organizational resilience: towards an integrated conceptual framework. J Bus Econ.

[19] CAPMAS (2021) https://www.data4sdgs.org/partner/capmas-central-agency-public-mobilization-and-statistics (accessed on 5th of July, 2021).

[20] Chen M, Liu H, Wei S, Gu J (2018). Top managers' managerial ties, supply chain integration, and firm performance in China: a social capital perspective. Ind Mark Manage.

[21] Chikerema L, Makanyeza C (2021). Enhancing the performance of micro-enterprises through market orientation: evidence from Harare. Zimbabwe Global Business Organiz Excel.

[22] Ciampi F, Demi S, Magrini A, Marzi G, Papa A (2021). Exploring the impact of big data analytics capabilities on business model innovation: The mediating role of entrepreneurial orientation. J Bus Res.

[23] Clauss T (2017). Measuring business model innovation: conceptualization, scale development, and proof of performance. RD Manag.

[24] Correia RJ, Dias JG, Teixeira MS (2020). Dynamic capabilities and competitive advantages as mediator variables between market orientation and business performance. J Strateg Manag.

[25] Dai B, Liang W (2022). The impact of big data technical skills on novel business model innovation based on the role of resource integration and environmental uncertainty. Sustainability.

[26] Didonet S, Simmons G, Díaz-Villavicencio G, Palmer M (2012). (2012) The relationship between small business market orientation and environmental uncertainty. Mark Intell Plan.

[27] Dijkstra TK, Henseler J (2015). Consistent and asymptotically normal PLS estimators for linear structural equations. Comput Stat Data Anal.

[28] Dobrovic J, Lambovska M, Gallo P, Timkova V (2018). Non-financial indicators and their importance in small and medium-sized enterprises. J Compet.

[29] Dong MC, Li CB, Tse DK (2013). Do business and political ties differ in cultivating marketing channels for foreign and local firms in China?. J Int Mark.

[30] D'souza C, Nanere M, Marimuthu M, Arwani M, Nguyen N (2021). Market orientation, performance and the mediating role of innovation in Indonesian SMEs. Asia Pacific J Market Logist.

[31] Eriksson T, Heikkilä M, Nummela N (2022). Business model innovation for resilient international growth. Small Enterp Res.

[32] Ezzi F, Jarboui A (2016). Does innovation strategy affect financial, social and environmental performance?. J Econ Finance Admin Sci.

[33] Farrukh M, Raza A, Waheed A (2021). Your network is your net worth: political ties and innovation performance. Eur J Innov Manag.

[34] Foss NJ, Saebi T (2017). Fifteen years of research on business model innovation: How far have we come, and where should we go?. J Manag.

[35] Gilmore A, Carson D (1999). Entrepreneurial marketing by networking. New England J Entrep.

[36] Gölgeci I, Kuivalainen O (2020). Does social capital matter for supply chain resilience? The role of absorptive capacity and marketing-supply chain management alignment. Ind Mark Manage.

[37] Gunawan T, Jacob J, Duysters G (2016). Network ties and entrepreneurial orientation: Innovative performance of SMEs in a developing country. Int Entrep Manag J.

[38] Hair JF, Risher JJ, Sarstedt M, Ringle CM (2019). When to use and how to report the results of PLS-SEM. Eur Bus Rev.

[39] Heider A, Gerken M, van Dinther N, Hülsbeck M (2021). Business model innovation through dynamic capabilities in small and medium enterprises–Evidence from the German Mittelstand. J Bus Res.

[40] Heikkilä M, Bouwman H, Heikkilä J (2017). From strategic goals to business model innovation paths: an exploratory study. J Small Bus Enterp Dev.

[41] Henseler J, Hubona G, Ray PA (2016). Using PLS path modeling in new technology research: updated guidelines. Ind Manag Data Syst.

[42] Hernández-Linares R, Kellermanns FW, López-Fernández MC (2021). Dynamic capabilities and SME performance: The moderating effect of market orientation. J Small Bus Manage.

[43] Ismail TH, El-Deeb M, Halim YT (2022). Do related party transactions affect the relationship between political connections and firm value?. Evidence Egypt Future Business J.

[44] Jaworski BJ, Kohli AK (1993). Market orientation: antecedents and consequences. J Mark.

[45] Julian CC, Mohamad O, Ahmed ZU, Sefnedi S (2014). The market orientation–Performance relationship: The empirical link in export ventures. Thunderbird Int Business Rev.

[46] Kafetzopoulos D, Psomas E, Skalkos D (2020). Innovation dimensions and business performance under environmental uncertainty. Eur J Innov Manag.

[47] Kasim A, Ekinci Y, Altınay L, Hussain K (2018). Impact of market orientation, organizational learning and market conditions on small and medium-size hospitality enterprises. J Hosp Market Manag.

[48] Khalil A, Abdelli MEA, Mogaji E (2022). Do digital technologies influence the relationship between the COVID-19 crisis and SMEs’ resilience in developing countries?. J Open Innov Technol Market Complex.

[49] Kolbe D, Frasquet M, Calderon H (2022). The role of market orientation and innovation capability in export performance of small-and medium-sized enterprises: a Latin American perspective. Multinatl Bus Rev.

[50] Laaksonen O, Peltoniemi M (2018). The essence of dynamic capabilities and their measurement. Int J Manag Rev.

[51] Latifi MA, Nikou S, Bouwman H (2021). Business model innovation and firm performance: Exploring causal mechanisms in SMEs. Technovation.

[52] Lin Y, Wu LY (2014). Exploring the role of dynamic capabilities in firm performance under the resource-based view framework. J Bus Res.

[53] Liu FH, Huang TL (2018). The influence of collaborative competence and service innovation on manufacturers' competitive advantage. J Business Indust Market.

[54] Ma Y, Wei H, Hu C, Jin C (2021). Research on the innovation path of business models based on the market orientation. Complexity.

[55] Miller K, McAdam M, Spieth P, Brady M (2021). Business models big and small: review of conceptualizations and constructs and future directions for SME business model research. J Bus Res.

[56] Miroshnychenko I, Strobl A, Matzler K, De Massis A (2021). Absorptive capacity, strategic flexibility, and business model innovation: Empirical evidence from Italian SMEs. J Bus Res.

[57] Najaf R, Najaf K (2021). Political ties and corporate performance: why efficiency matters?. J Business Socio-econ Develop.

[58] Naqshbandi MM (2016). Managerial ties and open innovation: examining the role of absorptive capacity. Manag Decis.

[59] Naqshbandi MM, Kaur S (2014). Do managerial ties support or stifle open innovation?. Ind Manag Data Syst.

[60] Narver JC, Slater SF, MacLachlan DL (2004). Responsive and proactive market orientation and new-product success. J Prod Innov Manag.

[61] Newman A, Prajogo D, Atherton A (2016). The influence of market orientation on innovation strategies. J Serv Theory Pract.

[62] Nitzl C, Roldan JL, Cepeda G (2016). Mediation analysis in partial least squares path modeling: Helping researchers discuss more sophisticated models. Ind Manag Data Syst.

[63] O'Donnell A (2004). The nature of networking in small firms. J Cetacean Res Manag.

[64] Ozanne LK, Chowdhury M, Prayag G, Mollenkopf DA (2022). SMEs navigating COVID-19: the influence of social capital and dynamic capabilities on organizational resilience. Ind Mark Manage.

[65] Panno A (2019). Performance measurement and management in small companies of the service sector; evidence from a sample of Italian hotels. Meas Bus Excell.

[66] Pucihar A, Lenart G, Kljajić Borštnar M, Vidmar D, Marolt M (2019). Drivers and outcomes of business model innovation—Micro, small and medium-sized enterprises perspective. Sustainability.

[67] Rajwani T, Liedong TA (2015). Political activity and firm performance within nonmarket research: A review and international comparative assessment. J World Bus.

[68] Randhawa K, Wilden R, Gudergan S (2021). How to innovate toward an ambidextrous business model? The role of dynamic capabilities and market orientation. J Bus Res.

[69] Salvato C, Vassolo R (2018). The sources of dynamism in dynamic capabilities. Strateg Manag J.

[70] Sami P, Rahnavard F, Alavi Tabar A (2019). The effect of political and business ties on firm performance. Manag Res Rev.

[71] Sarwar Z, Khan MA, Yang Z, Khan A, Haseeb M, Sarwar A (2021). An investigation of entrepreneurial SMEs’ network capability and social capital to accomplish innovativeness: a dynamic capability perspective. SAGE Open.

[72] Schilke O (2014). On the contingent value of dynamic capabilities for competitive advantage: The nonlinear moderating effect of environmental dynamism. Strateg Manag J.

[73] Sheng S, Zhou KZ, Li JJ (2011). The effects of business and political ties on firm performance: evidence from China. J Mark.

[74] Shirodkar V, Mohr AT (2015). Explaining foreign firms’ approaches to corporate political activity in emerging economies: the effects of resource criticality, product diversification, inter-subsidiary integration, and business ties. Int Bus Rev.

[75] Teece DJ (2018). Business models and dynamic capabilities. Long Range Plan.

[76] Thongsri N, Chang AKH (2019). Interactions among factors influencing product innovation and innovation behaviour: Market orientation, managerial ties, and government support. Sustainability.

[77] Tian H, Iqbal S, Anwar F, Akhtar S, Khan MAS, Wang W (2021). Network embeddedness and innovation performance: a mediation moderation analysis using PLS-SEM. Bus Process Manag J.

[78] Tutar H, Nart S, Bingöl D (2015). The effects of strategic orientations on innovation capabilities and market performance: The case of ASEM. Procedia Soc Behav Sci.

[79] Vij S, Bedi HS (2016). Are subjective business performance measures justified?. Int J Product Perform Manag.

[80] Wang CL, Chung HF (2013). The moderating role of managerial ties in market orientation and innovation: An Asian perspective. J Bus Res.

[81] Wang Y, Zeng D, Di Benedetto CA, Song M (2013). Environmental determinants of responsive and proactive market orientations. J Business Ind Market.

[82] Wang D, Guo H, Liu L (2017). One goal, two paths: How managerial ties impact business model innovation in a transition economy. J Organ Chang Manag.

[83] Wei Z, Zhao J, Zhang C (2014). Organizational ambidexterity, market orientation, and firm performance. J Eng Tech Manage.

[84] Wilden R, Gudergan S, Lings I (2019). The interplay and growth implications of dynamic capabilities and market orientation. Ind Mark Manage.

[85] Yan H, He X, Cheng B (2017). Managerial ties, market orientation and export performance. Manag Organ Rev.

[86] Yang D, Wei Z, Shi H, Zhao J (2020). Market orientation, strategic flexibility and business model innovation. J Business Ind Market.

[87] Zaazou ZA, Abdou DS (2021). Egyptian small and medium sized enterprises’ battle against COVID-19 pandemic: March–July 2020. J Human Appl Soc Sci.

[88] Zhang D, Linderman K, Schroeder RG (2012). The moderating role of contextual factors on quality management practices. J Oper Manag.

[89] Zhang M, Qi Y, Wang Z, Zhao X, Pawar KS (2019). Effects of business and political ties on product innovation performance: Evidence from China and India. Technovation.

[90] Zheng W, Singh K, Mitchell W (2015). Buffering and enabling: The impact of interlocking political ties on firm survival and sales growth. Strateg Manag J.

[91] Zhu H, Chung CN (2014). Portfolios of political ties and business group strategy in emerging economies: Evidence from Taiwan. Adm Sci Q.

[92] Zott C, Amit R, Massa L (2011). The business model: recent developments and future research. J Manag.

